# Angiogenesis Is Induced and Wound Size Is Reduced by Electrical Stimulation in an Acute Wound Healing Model in Human Skin

**DOI:** 10.1371/journal.pone.0124502

**Published:** 2015-04-30

**Authors:** Sara Ud-Din, Anil Sebastian, Pamela Giddings, James Colthurst, Sigrid Whiteside, Julie Morris, Richard Nuccitelli, Christine Pullar, Mo Baguneid, Ardeshir Bayat

**Affiliations:** 1 Plastic & Reconstructive Surgery Research, Manchester Institute of Biotechnology, University of Manchester, Manchester, United Kingdom; 2 University Hospital of South Manchester NHS Foundation Trust, Institute of Inflammation and Repair, Faculty of Medical and Human Sciences, University of Manchester, Manchester Academic Health Science Centre, Manchester, United Kingdom; 3 Oxford Bioelectronics, Innovation Centre, Abingdon, United Kingdom; 4 Medical Statistics, University Hospital South Manchester, Manchester, United Kingdom; 5 BioElectroMed Corporation, Burlingame, CA, United States of America; 6 Department of Cell Physiology and Pharmacology, University of Leicester, Leicester, United Kingdom; 7 Centre for Dermatology Research, Institute of Inflammation and Repair, University of Manchester, Manchester, United Kingdom; University of New Mexico HSC, UNITED STATES

## Abstract

Angiogenesis is critical for wound healing. Insufficient angiogenesis can result in impaired wound healing and chronic wound formation. Electrical stimulation (ES) has been shown to enhance angiogenesis. We previously showed that ES enhanced angiogenesis in acute wounds at one time point (day 14). The aim of this study was to further evaluate the role of ES in affecting angiogenesis during the acute phase of cutaneous wound healing over multiple time points. We compared the angiogenic response to wounding in 40 healthy volunteers (divided into two groups and randomised), treated with ES (post-ES) and compared them to secondary intention wound healing (control). Biopsy time points monitored were days 0, 3, 7, 10, 14. Objective non-invasive measures and H&E analysis were performed in addition to immunohistochemistry (IHC) and Western blotting (WB). Wound volume was significantly reduced on D7, 10 and 14 post-ES (p = 0.003, p = 0.002, p<0.001 respectively), surface area was reduced on days 10 (p = 0.001) and 14 (p<0.001) and wound diameter reduced on days 10 (p = 0.009) and 14 (p = 0.002). Blood flow increased significantly post-ES on D10 (p = 0.002) and 14 (p = 0.001). Angiogenic markers were up-regulated following ES application; protein analysis by IHC showed an increase (p<0.05) in VEGF-A expression by ES treatment on days 7, 10 and 14 (39%, 27% and 35% respectively) and PLGF expression on days 3 and 7 (40% on both days), compared to normal healing. Similarly, WB demonstrated an increase (p<0.05) in PLGF on days 7 and 14 (51% and 35% respectively). WB studies showed a significant increase of 30% (p>0.05) on day 14 in VEGF-A expression post-ES compared to controls. Furthermore, organisation of granulation tissue was improved on day 14 post-ES. This randomised controlled trial has shown that ES enhanced wound healing by reduced wound dimensions and increased VEGF-A and PLGF expression in acute cutaneous wounds, which further substantiates the role of ES in up-regulating angiogenesis as observed over multiple time points. This therapeutic approach may have potential application for clinical management of delayed and chronic wounds.

## Introduction

Angiogenesis is a critical component for processes in wound healing and is defined as the formation of new capillaries from pre-existing blood vessels [1, 2]. Insufficient angiogenesis can result in impaired wound healing and chronic wound formation [4–8]. Electrical stimulation (ES) in its various forms has been shown to enhance wound healing by promoting the migration of keratinocytes and macrophages, enhancing angiogenesis, stimulating fibroblasts, and influencing protein synthesis throughout the inflammatory, proliferative and remodelling phases of healing [9–11]. Electrical signals have been shown to stimulate angiogenesis and organise blood vessel formation [3]. There is limited information on the influence of ES on angiogenesis after acute wounding in human skin, as most research is restricted to animal models. Animal studies have shown that angiogenesis is induced by ES in ischemic and non-ischemic rat limbs, and is facilitated by the increased expression of VEGF in muscle cells [12,13]. Endogenous electric fields are able to direct the migration of epithelial cells during wound healing and may contribute to regulation of angiogenesis [14].

Previous studies have shown that certain electrical currents, such as direct and alternating currents, are useful in treating; diabetic foot ulcers, skin ulcers and chronic wounds [15,16]. We have investigated the in vitro effect of different types of ES on the expression of collagen in skin fibroblasts. Importantly, we highlighted the role of a novel waveform termed degenerate wave (DW is a degenerating sine wave, which deteriorates over time) and demonstrated its beneficial effects compared to other known waveforms such as direct and alternating currents [17]. In a recent in vivo study, we also demonstrated the application of an ES biofeedback device, which produces DW in a human volunteer study (n = 20) involving only one time point (day 14) punch biopsy [18]. The results showed increased blood flow in acute cutaneous wounds following the biopsy compared to controls that had not received ES. Additional experimental gene and protein studies corroborated these findings by demonstrating up-regulation of angiogenesis and down-regulation of inflammation in ES treated wounds [19]. However, this study [18,19] only evaluated the effects of ES at one time point (day 14), with 20 participants and using only two objective measures.

In view of the above scientifically interesting and clinically relevant findings, the aim of this study was to further evaluate the role of ES in affecting angiogenesis during the acute phase of cutaneous wound healing over multiple time points to identify if the enhanced effect occurred earlier than day 14. In addition to multiple time points, we also used an increased number of sequential skin biopsies, increased number of participants (n = 40) with randomisation verified using additional non-invasive objective devices such as a three-dimensional camera and a Dermacorder (BioElectromed Corporation, USA).

## Methods

### Ethics Statement

Healthy participants were enrolled into the study at the University Hospital of South Manchester NHS Foundation Trust, England, UK. South Manchester Research Ethics Committee and Trust Research and Development department approval were granted for the study (Ethics number: 09/H1012/3). If suitable (inclusion and exclusion criteria outlined in S1 Table), participants were asked to provide written consent to take part and were then enrolled in the study. All study participants provided written consent.

### Demographics

#### Cohort 1

Twenty participants were recruited onto cohort 1, the majority of which were female (n = 14, 70%). Most participants were between the ages of 19–24 years and all were of Caucasian ethnicity (n = 40, 100%). Most participants had a Fitzpatrick skin type of II (fair skin, tans poorly, burns easily) (n = 9, 45%) or skin type III (fair skin, tans moderately, sometimes burns) (n = 9, 45%). Furthermore, all participants were right handed.

#### Cohort 2

Twenty participants were recruited onto cohort 2, the majority of which were female (n = 15, 75%). Most participants were between the ages of 25–30 years and all were of Caucasian ethnicity (n = 40, 100%). Most participants had a Fitzpatrick skin type III (fair skin, tans moderately, sometimes burns) (n = 10, 50%). Furthermore, all participants were right handed. Demographic data for both cohorts are outlined in S2 Table.

### Study Design

**Fig 1:**
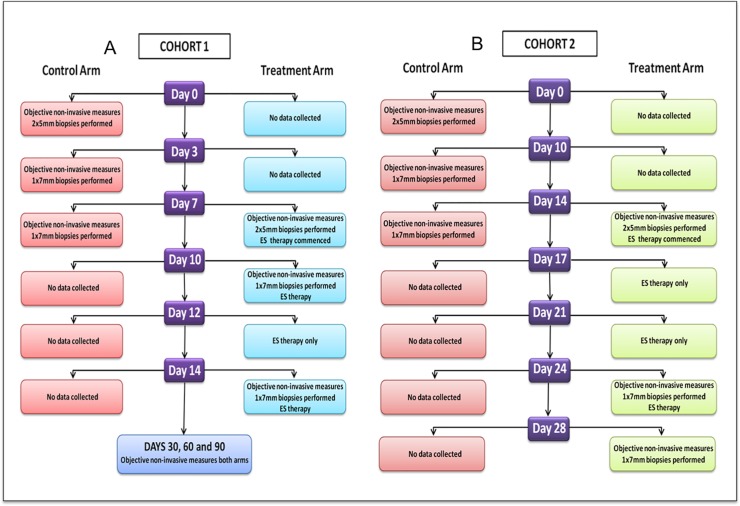
Flowcharts outlining the study methodology. A. A flowchart outlining the methodology of the study for cohort 1. Time points for this cohort were at days 0, 3, 7, 10, 12, 14, 30, 60 and 90. B. A flowchart outlining the methodology for the study for cohort 2. Time points for this cohort were at days 0, 10, 14, 17, 21, 24 and 28.

Forty healthy volunteers were split equally into two groups: Cohort 1 and Cohort 2 (Fig 1). The rationale for including two cohorts was to look at multiple time points. This would not have been possible in one cohort to look at day 0, 3, 7, 10 and 14 due to the large number of punch biopsies to be performed in each participant.

#### Cohort 1

**Fig 2:**
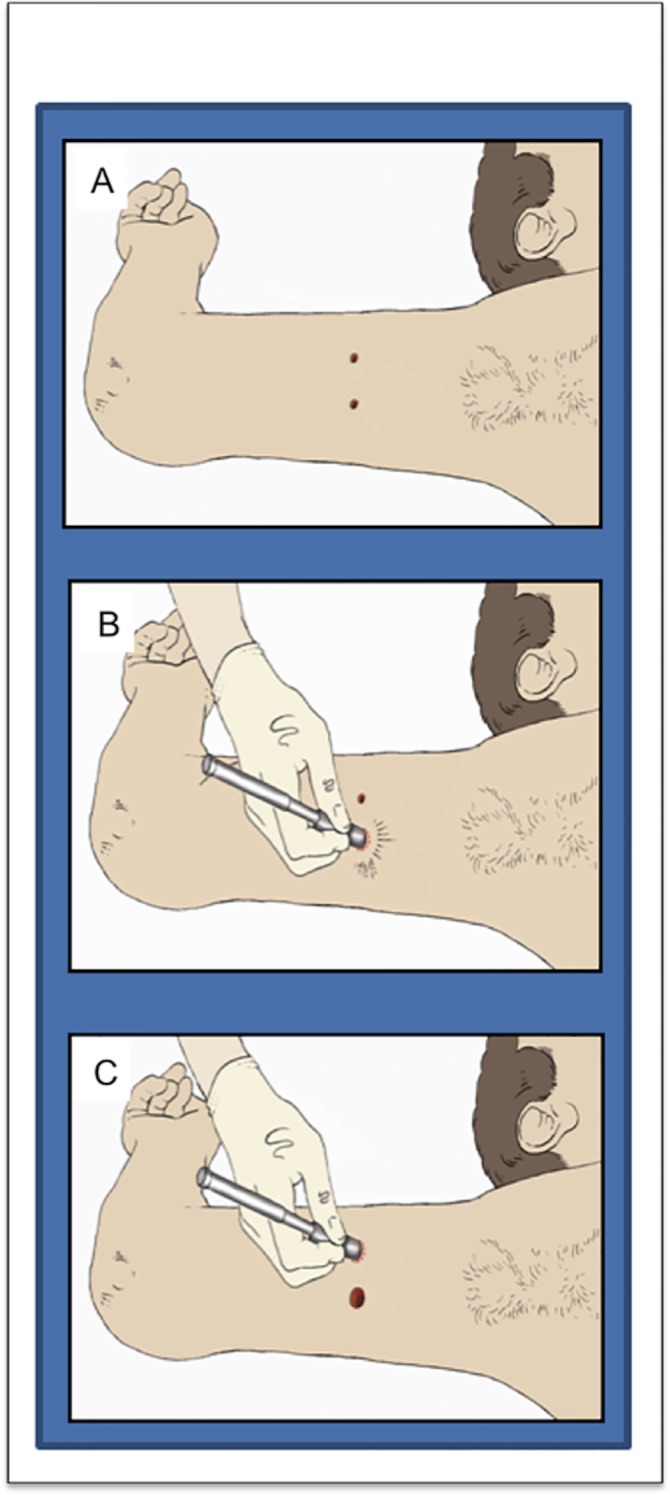
Illustrations to demonstrate the punch biopsy method. A. Two 5mm punch biopsies were performed in the inner upper arm. B. One 7mm punch biopsy was used to excise one of the previous biopsy sites. C. A further 7mm punch biopsy was performed to encompass the second previous biopsy site.

On day 0, participants had two 5mm diameter full thickness skin biopsies performed under local anaesthetic (0.5% Bupivacaine) in their upper inner arm which acted as the control arm to monitor normal wound healing. Full thickness was defined as removing the entire epidermis and dermis to expose hypodermic fat. Additionally, the anaesthetic used has been utilised routinely following surgery and there has been no evidence, which suggests this could have any detrimental effects on angiogenesis or wound healing. The punch biopsies were performed 5cm from the axillary hairline and parallel to the medial epicondyle and were 2.5cm in distance from each other (Fig 2). Pressure was applied to each biopsy site until haemostasis had been achieved. Participant arms were also randomised to allocate for handedness and to avoid anatomical side-specific effects.

On day 3, one 7 mm diameter skin biopsy was performed encompassing one of the previous wound sites. On day 7, another 7 mm diameter skin biopsy was performed to excise the second previous wound site. On the same day, the process was repeated in the same anatomical location (on the corresponding days 7, 10 and 14) but on the contralateral arm and an intervention stage of ES was instigated to the second arm (on days 7, 10, 12 an 14). This cohort also had follow-up time points on days 30, 60 and 90 in both arms. Objective measurements were taken before the application of ES at each visit.

#### Cohort 2

Cohort 2 followed the same process as Cohort 1 but at different biopsy time points, days 0, 10, 14 (control arm) and corresponding days 14, 24 and 28 (post-ES arm). ES was applied on days 14, 17, 21 and 24. Additionally, objective non-invasive measures were performed at each visit for all participants in both cohorts and were taken before any intervention or measurements at each visit.

### Wound site care

The biopsy wound sites were dressed with Kaltostat (ConvaTec, Middlesex, UK), gauze and Tegaderm + pad dressing (3M, Minnesota, USA). Participants were asked to ensure the dressings remained *in situ* for 48 hours only and then no further dressings were required and wounds were left exposed to the air for the rest of the study. Additionally, all wounds were monitored at each visit. No sutures were required following the biopsy procedures for any of the participants.

### Electrical Stimulation Device

The electrical stimulation device used was the Fenzian system (Fenzian Ltd, Hungerford, UK) which is Food and Drug Administration (FDA) 510(k) registered and Conformite Europenne (CE) approved. It is a transcutaneous low intensity device, which detects changes in skin impedance. This device forms part of a biofeedback link with the individual’s normal physiological repair. This modality follows the theory that the normal electrical potential of skin forms a global electrical network, which reflects the underlying neurological activity through changes in skin impedance. Using a concentric electrode the device detects the skin’s electrical impedance and adjusts the outgoing microcurrent electrical biofeedback impulses. The device produces the degenerate waves and delivers 0.004 milliamps, 20–80V, has a frequency default of 60Hz and impulses, which last approximately six-hundredth of a second. ES therapy was administered for all participants in both cohorts to the arms on 4 occasions. For cohort 1, ES was performed on days 7, 10, 12 and 14. For cohort 2, ES was administered on days 14, 17, 21 and 24. Electrical stimulation was applied by a single researcher and administered according to a standardised local protocol around the wound site. Treatments were performed for approximately 30 minutes. The intensity of the device was comfortable for the participant and the treatment was painless.

### Objective Non-invasive Measures

Objective non-invasive imaging modalities were used at each time point for both cohorts prior to any intervention to monitor the progression of wound healing which will be outlined below.

#### Full-field Laser Perfusion Imaging

Full-field Laser Perfusion Imaging (FLPI) (Moor Instruments Ltd, Axminster, UK) is a technique, which measures blood flow in the skin’s microcirculation. This device uses low power light from a monochromatic stable laser and this is applied to a tissue, which becomes scattered by moving red blood cells which broadens the frequency. This is then photo detected and is processed to provide a blood flow measurement. Blood flow known as “flux” is described in arbitrary “perfusion units”. This is proportional to the speed and concentration of red blood cells in the tissue [20]. This device has been used in an acute human wound model and demonstrated that this technique is useful for small acute wounds [21].

#### Three-Dimensional (3D) Camera

The 3D LifeViz camera (Quantificare S.A., Cedez, France) in conjunction with its software system provides 3D visualisation with quantitative measurement [22]. The DermaPix image management software enables analysis of the images. This device was used to provide accurate imaging and measurements of the volume, surface area, and diameter of all participants’ biopsy sites as well as the depth of the wounds. 3D imaging has also been used to quantitate the surface roughness of skin and scars in a study by Bloemen et al [23]. They found that the inter-observer reliability was satisfactory.

#### Dermacorder

The Dermacorder (BioElectromed Corporation, USA) is a bioelectric field imager used for measuring the electrical field (EF) in human skin [24]. It is thought that skin has a transepidermal potential of approximately 10–60mV [25]. In addition to the polarised distribution of ion channels, the distribution of Na^+^/K^+^ ATPase ion pumps is also highly localised [25]. When a wound is made, a low resistance pathway is formed and the transepidermal potential reaches 0mV at the wound. However, at distances of 1mm away or greater, the potential remains normal [24]. This drives a current of injury out of the wound and generates a lateral EF that can be detected non-invasively using the Dermacorder. The use of this device has been verified in an acute human skin wound study by Nuccitelli et al [26]. We used this device to measure the EF at the edges of the biopsy wounds on half of the patients (n = 20) due to availability of the device.

#### RNA isolation, cDNA synthesis and quantitative real time- polymerase chain reaction

For *in vivo* biopsies obtained on different days of healing, RNA isolation, cDNA synthesis, and qRT-PCR were performed as explained previously [27]. Briefly, after tissues were diced, 0·5–1 ml Trizol was added and the tubes were introduced to Qiagen tissue lyser (Qiagen, Crawley, UK) to homogenization. Additionally, it was centrifuged and the resulting supernatant was mixed with 0·2 ml chloroform (cat. 10050090; Fisher Scientific, Loughborough, UK). The mixture was then spun to get the upper aqueous layer and mixed with an equal volume of 70% ethanol, which was then further processed with RNeasy kit (cat. 74106; Qiagen) according to the manufacturer’s instructions to extract total RNA. DNase treatment was then carried out using DNA-free kit (cat. 79254; Life Technologies, Paisley, UK) according to the manufacturer’s protocol. qScript cDNA SuperMix (cat. 95048; Quanta Biosciences, Gaithersburg, MD, USA) was used for cDNA synthesis. qRT-PCR was done in LightCycler 480 II platform (Roche Diagnostics, Burgess Hill, UK). RPL32 was used as the internal control and ΔΔC_T_ method was used to calculate fold change of gene expression. The primers used are detailed in S3 Table.

### Tissue processing and histological analysis

#### Immunohistochemistry

Tissues (biopsies from human volunteers) at different time points of healing were initially collected in neutral-buffered formalin (cat. F5304; Sigma-Aldrich, Dorset, UK). They were fixed for 3–4 days in formalin at 4°C and later, they were wax-embedded. Tissue sections were further analysed for Hematoxylin and Eosin (H&E) staining. Briefly, the sections were de-waxed in a series of xylene (cat. X/0200/17; Fisher Scientific, Loughborough, UK) for 25 min and rehydrated in graded ethanol (100% to 50%; cat. E/06500F/17; Fisher Scientific, Loughborough, UK). They were washed in water and stained for nuclei with Harris haematoxylin (cat. 72711; Thermo Scientific, Loughborough, UK) for 10 min and washed with tap water. The tissues were differentiated in 0.5% acid-alcohol solution and washed again. They were counter-stained with Eosin Y (cat. 6766007; Thermo Scientific) for 1 min and washed. Later, sections were dehydrated in graded ethanol (95% and 100%), cleared with xylene and mounted on slides. Immunohistochemical staining was performed as detailed in our previous report [27]. Briefly, dewaxing was done in the same way as explained for H&E staining. Antigen retrieval was done with citrate buffer (pH 6.0) at 60°C for 1 h and then followed Novolink polymer detection kit protocol (cat. RE7150-K, Leica Biosystems Newcastle ltd., Newcastle Upon-Tyne, UK) which included endogenous peroxidase blocking for 30 min, protein blocking for 30 min and further incubation with primary and secondary (post primary) antibodies. After Novolink polymer incubation, sections were incubated with the substrate/chromogen, 3,3’ - diaminobenzidine (DAB), prepared from DAB chromogen and Novolink DAB substrate buffer (Polymer). Sections were counterstained with Hematoxylin and cover-slipped. Antibodies, incubation time and detection methods are detailed in S4 Table.

#### Immunohistochemistry image analysis by Definiens software

Definiens tissue studio version 3.51 (Definiens AG, Munchen, Germany) was used to quantitate the IHC results. Initially, in the cellular analysis module, 12 subsets were selected in the tissue specimen for site recognition. Nuclear detection, nuclear area detection and nucleus classification were also done on the subsets. For IHC-peroxidase staining, hematoxylin threshold was adjusted for cell detection. Analysis was done in 20X magnification.

#### Western blotting (WB)

Tissues were retrieved from liquid nitrogen, diced into very small pieces using a clean razor blade and mashed properly with a mortar and pestle. They were lysed in RIPA buffer supplemented with protease and phosphatase inhibitor cocktail. The tissues were then homogenised in a tissue lyser (Qiagen, UK) at 30 oscillations s^−1^ for 10 min. Subsequently, they were incubated in ice for 30 min. Later, they were centrifuged at 10,000 *g* for 20 min and supernatant obtained was the total tissue lysate. Protein estimation was performed with Non-interfering protein assay kit (cat. 488250; Merck, Middlesex, UK). Equal amount of proteins (30 μg) were denatured and resolved on 4 to 12% Bis-Tris gels (cat. NP0321; Life Technologies, Paisley, UK) and electrophoresed according to the manufacturer's instructions. The proteins were blotted on polyvinylidene difluoride membranes (PVDF, cat. IB401001; Life Technologies) from polyacrylamide gels using iBlot Dry Blotting System (Life Technologies). Membranes were incubated for 1 h at room temperature in Odyssey blocking buffer (cat. 927–40000; LI-COR Biosciences, Cambridge, UK). After the blocking step, membranes were incubated overnight at 4°C with the respective primary antibodies, followed by the secondary antibody incubation for 1 hour at 37°C. 1-step nitro blue tetrazolium / S-bromo-4-chloroindoxyl phosphate (NBT/BCIP, cat. 34042; Thermo Scientific) was used to develop the blot. Antibodies, incubation time and detection methods are detailed in S5 Table.

### Statistical Analysis

Due to the variability and skewness of the data distributions, non-parametric summaries of median and range (minimum, maximum) were utilised. Additionally, measurements for the control arm and post-ES arm were compared on all wound days using pair-wise Wilcoxon signed ranks tests. All analyses used a two-sided 1% significance level; adjusting for repeated comparisons on multiple study days using Bonferroni adjustment. For cohort 1, wound days 30, 60 and 90 were considered to correspond to post-ES arm wound days 23, 53 and 83. At these follow-up time points, there was no control arm. The data were analysed to identify any trends or assess any effects that had been sustained in the treated arm. The randomisation was conducted by the statisticians in nQuery Advisor 7.0 using a computer generated permuted block design with mixed block sizes and random seed. All summaries and analyses were performed using SPSS (version 20; IBM Corporation, Armonk, NY, USA). For histological experiments, data is presented as mean +/- standard deviation from three independent experiments performed in triplicates (n = 3). Statistical analysis was calculated using one way ANOVA for comparison between three groups with Tukey post hoc test, and student’s *t* test for comparison between two groups. Confidence intervals of 95% with corresponding p value of 0.05 was chosen throughout analysis. Data collection and analysis were not blinded as a single researcher performed all aspects of the study except statistical analysis, which was performed by independent statisticians. Additionally, the histological analysis was performed by a scientist at the laboratory.

## Results

The non-invasive clinical results will now be presented in relation to wound surface area, wound volume, wound diameter, wound depth and blood flow. Additionally, the histological analysis results will also be presented.

### Non-invasive clinical results

#### Three dimensional imaging analysis showed a significant reduction in wound surface area on days 10, 14, 30, 60 and 90 after electrical stimulation

**Fig 3:**
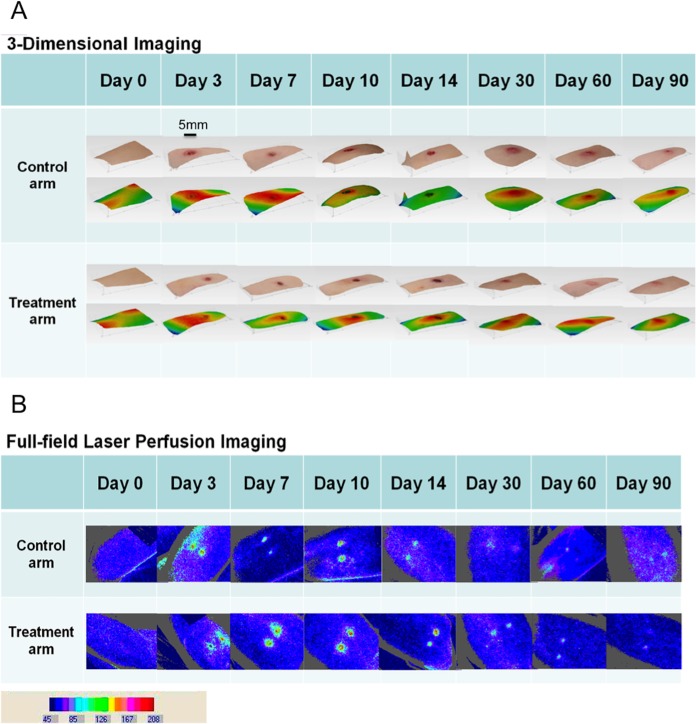
Images produced from the non-invasive imaging devices. A. Images from the 3-dimensional camera are shown which demonstrate the size of the biopsy wound sites are smaller post electrical stimulation (ES) compared to the control. The normal wound image is displayed with the difference in heights images corresponding shown below. The difference in heights images show the highest points are indicated in red and yellow, whilst the lowest points are displayed as green and blue. B. Images from the full-field laser perfusion imager at each time point demonstrating the blood flow for the control and post-ES arm. The differences in blood flow (flux) in the biopsy sites are depicted by the change of colour intensity in the images.

Wound surface area was less in the post-ES arm compared to the control arm at all time points (Fig 3A and S6 Table). However, there were no statistically significant differences for surface area on days 3 and 7 when comparing the control arm to the post-ES arm. At day 10 wound surface area in the control arm was generally higher with median of 9.21 (quartiles 7.64 and 10.70) (quartiles are displayed as lower quartile followed by upper quartile throughout); whereas for the post-ES arm surface area measurements were significantly lower with median of 7.22 (quartiles 5.11 and 8.88) (p = 0.001). There was a 21.6% decrease in surface area post-ES on day 10. On day 14, surface area measurements in the control arm were generally higher with a median of 7.70 (quartiles 5.95 and 10.13); whereas post-ES the measurements were mostly lower with a median of 4.49 (quartiles 3.58 and 5.87) (p<0.001). There was a 41.7% decrease in surface area post-ES on day 14. Surface area was also statistically significantly reduced post-ES on day 30 (p = 0.004), day 60 (p = 0.001) and day 90 (p = 0.003). There was a 16.4% decrease on day 30, a 31.1% decrease on day 60 and a 30% decrease on day 90.

#### Three dimensional imaging analysis showed a significant reduction in wound volume on days 7, 10 and 14 after electrical stimulation

Wound volume was lower in the post-ES arm compared to the control arm at all time points (Fig 3A and S7 Table). Control arm measurements for wound volume on day 7 were generally higher with a median of 1.98 (quartiles 1.50 and 2.44); whereas post-ES volume measurements were significantly lower with a median of 1.21 (quartiles 0.56 and 1.95) (p = 0.003). There was a 38.9% decrease in wound volume post-ES on day 7. On day 10, the volume in the control arm was higher with a median of 2.48 (quartiles 1.78 and 3.81); whereas post-ES, the volume was significantly lower with a median of 1.54 (quartiles 1.26 and 2.77) (p = 0.002). There was a 37.9% decrease in wound volume post-ES on day 10. On day 14, wound volume was higher with a median of 2.04 (quartiles 1.45 and 3.31); whereas post-ES wound volume was significantly lower with a median of 1.14 (quartiles 0.69 and 1.34) (p<0.001). There was a 44.1% decrease in wound volume post-ES on day 14.

#### Three dimensional imaging analysis demonstrated a statistically significant reduction in wound diameter on days 10, 14 and 90 after electrical stimulation

Wound diameter was less in the post-ES arm compared to the control arm at all time points (Fig 3A and S8 Table). Although, there were no statistically significant differences in diameter size on days 3, 7, 30 and 60 when comparing the control to the post-ES arm. However, wound diameter was significantly less on days 10, 14 and 90. On day 10, control arm measurements for wound diameter were higher with a median of 4.32 (quartiles 3.98 and 4.81); whereas post-ES the diameter was significantly less with a median of 3.84 (quartiles 3.21 and 4.45) (p = 0.009). There was an 11.1% decrease in wound diameter post-ES on day 10. On day 14, control measurements for wound diameter were increased with a median of 3.75 (quartiles 3.39 and 4.45); whereas post-ES measurements were significantly reduced with a median of 3.15 (quartiles 2.82 and 3.44) (p = 0.002). There was a 16% decrease in wound diameter post-ES at day 14. On day 90, wound diameter was significantly reduced post-ES compared to controls (p = 0.007). There was a 16.2% decrease in diameter post-ES on day 90.

#### Three dimensional imaging analysis demonstrated reduced wound depth measurements after electrical stimulation

Wound depth measurements were less in the post-ES arm compared to the control arm at all time points although not significantly (Fig 3A and S9 Table). Wound depth measurements remained approximately similar across all time points for both arms (day 3; p = 0.260, day 7; p = 0.048, day 10; p = 0.390, day 14; p = 0.341, day 30; p = 0.732, day 60; p = 0.040, day 90; p = 0.014).

#### Electrical field (EF) measurements were higher after electrical stimulation

EF measurements at the wound edges were mostly increased in the post-ES arm on days 3 (p = 0.050), 10 (p = 0.028), 14 (p = 0.012) and 30 (p = 0.169) compared to the control arm and were reduced in the post-ES arm on days 60 and 90 (Table 1). Although, there were no statistically significant differences noted. At day 3, EF measurements demonstrated a 62% increase in the post-ES arm. At day 10, EF measurements showed a 47% increase in the post-ES arm compared to the control. At day 14, there was a 79% increase in the EF post-ES compared to the control arm.

**Table 1:** Electrical Field (mV/mm): Median (Range) for biopsy arms and differences in biopsy arms.

Day	N	Control Arm	Post-ES Arm	Difference of control vs. post-ES	P-value
Day 010					
Day 3	8	140.0 (50.72,240.0)	226.88 (81.30, 398.2)	67.95 (-84.7, 184.28)	0.050
Day 7	4	230.25 (178.0, 278.2)	255.0 (213.2, 380.0)	18.50 (-15.5, 165.0)	-
Day 10	6	153.8 (88.8, 216.8)	225.5 (178.0, 283.1)	70.1 (46.6, 130.2)	0.028
Day 14	8	161.3 (80.0, 299.4)	288.2 (117.0, 385.8)	75.5 (18.25, 231.0	0.012
Day 30	10	181.25 (125, 291.6)	202.9 (120.6, 380)	37.3 (-95.0, 100.4)	0.169
Day 60	4	169.2 (120.0, 185.8)	140.45 (95.0, 222.1)	-10.65 (-90.80, 65.9)	-
Day 90	2	129.45 (96.0, 162.9)	84.5 (84.0, 85.0)	-44.95 (-77.9, -12.0)	-

Difference: Post ES—control

p-values from unadjusted paired Wilcoxon Signed Ranks Test

#### Full-field laser perfusion imaging demonstrated a statistically significant increase in blood flow on days 10 and 14 after electrical stimulation

There were no statistically significant differences for blood flow on days 3, 7, 30, 60 and 90 when comparing changes from normal skin to the wound site. However, blood flow was statistically significantly increased in the post-ES arm on days 10 and 14 (Fig 3B and S10 Table). On day 10, blood flow was lower in the control arm with a median of 82.30 (quartiles 62.70 and 162.38), compared to blood flow, which was significantly higher in the post-ES arm with a median of 168.35 (quartiles 130.78 and 223.48) (p = 0.002). There was 104.5% increase in blood flow post-ES on day 10. On day 14, blood flow was lower in the control arm with a median of 77.90 (quartiles 39.18 and 105.98), whereas blood flow was increased in the post-ES arm with a median of 139.15 (quartiles 101.38 and 208.75) (p = 0.001). There was a 78.6% increase in blood flow post-ES on day 14.

**Fig 4:**
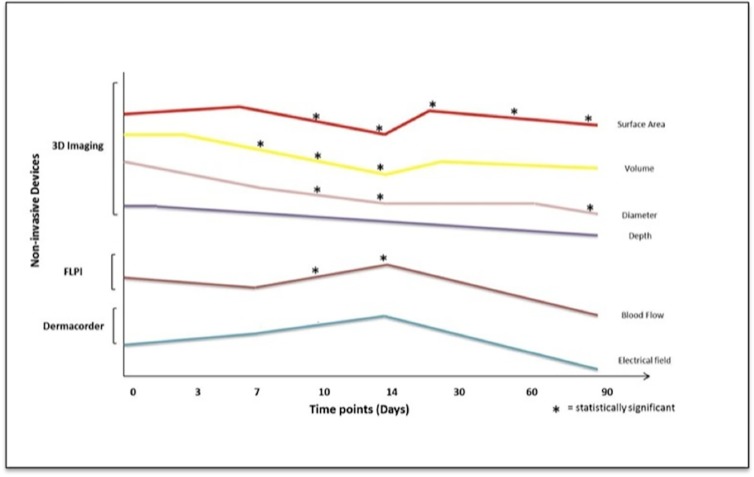
A graph demonstrating a representation of the trends noted. A graph collating the results from this electrical stimulation (ES) temporal punch biopsy study which displays a representation of the trends found for all of the time points combined. Each line represents the trend for the post-ES arm for each parameter and displays the points at which these were statistically significant. The different objective non-invasive devices, which were used are shown on the y-axis and the measurements taken for each are presented by a different colour line on the graph. The statistical significances found are highlighted on the graph (* = statistical significance). 3-dimensional (3D) imaging showed that the wound surface area was significantly lower on days 10, 14, 30 60 and 90 following the application of ES. Wound volume was significantly reduced post-ES on days 7, 10 and 14. Wound diameter was significantly lower on days 10, 14 and 90 following ES. Full-field laser perfusion imaging (FLPI) demonstrated a significant increase in blood flow at days 10 and 14 following the application of ES. Furthermore, the Dermacorder showed that the electrical field (EF) at the wound edges were increased post-ES although not significantly.

All of the aforementioned *in vivo* data are summarised in Fig 4.

### Histological Analysis Results

#### Histological analysis demonstrated improved organisation of granulation tissue on day 14 following electrical stimulation

**Fig 5:**
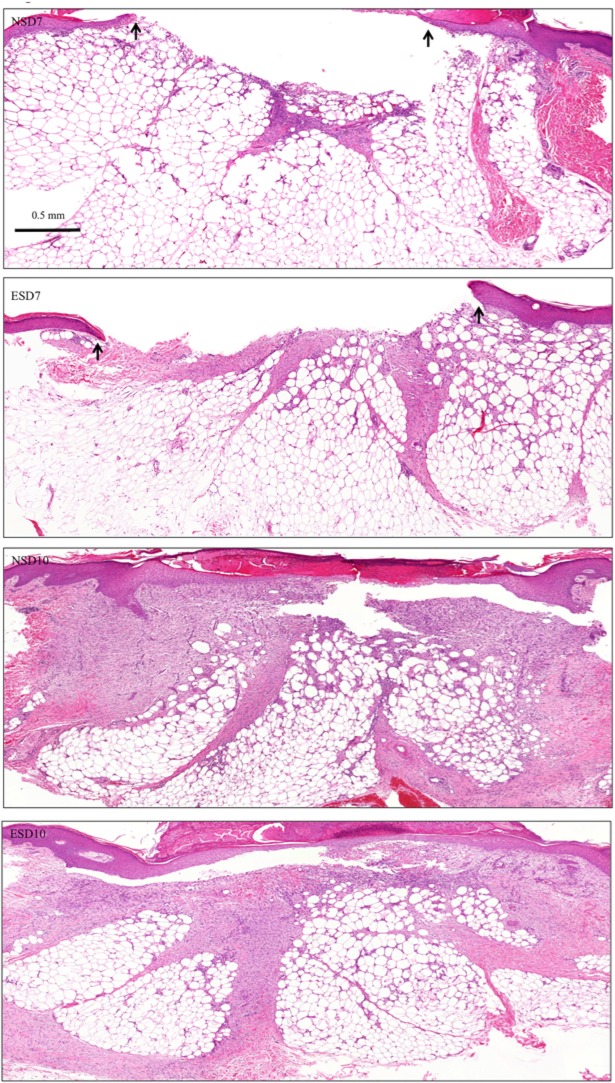
Hematoxylin and Eosin staining (healing day 7 and 10). Hematoxylin and Eosin staining on days 7 and 10 on normal healing tissues and corresponding ES treated healing tissues.

**Fig 6:**
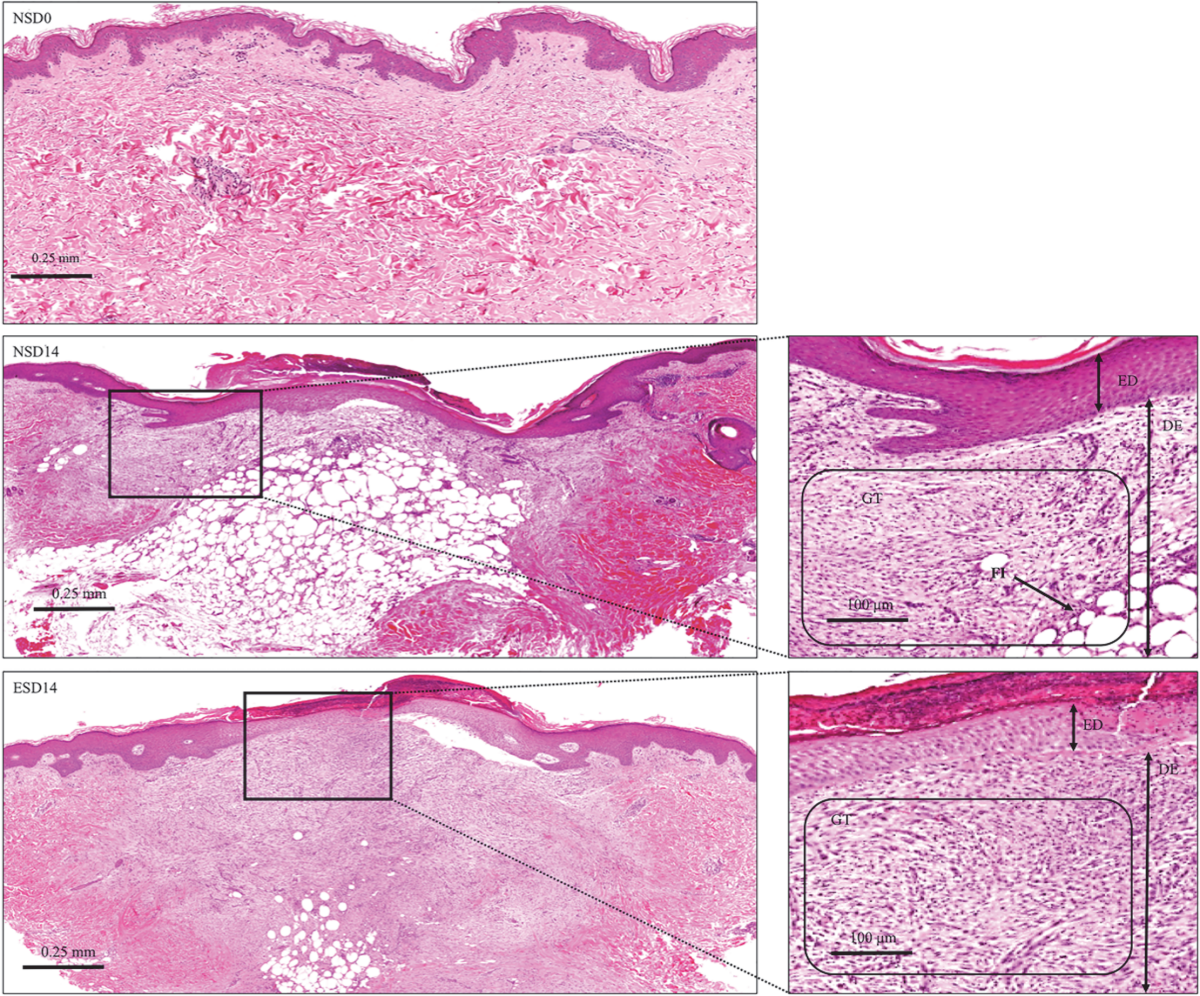
Hematoxylin and Eosin staining (normal skin and healing day 14). Hematoxylin and Eosin staining on days 0 (NSD0 or normal skin) and 14 of normal healing tissues (NSD14) and ES treated healing tissues (ESD14). ES samples showed a similar phenotype to normal skin with better wound bed re-organisation and accelerated granulation tissue stage development. ED is epidermis, DE is dermis, GT is granulation tissue, FT is fat/adipose tissue.

Histomorphometric progression of cutaneous wound healing in sequential biopsies were evaluated using H&E staining. All tissue samples (both post-ES and control) on day 3, did not show any morphological difference of note. However, on days 7 and 10, the granulation area was increased and epidermal organisation was markedly improved in post-ES samples (Fig 5). Post-ES samples on day 14 (ESD14) clearly showed increased granulation tissue area and decreased fat intrusion into the granulation surface, compared to control normal skin samples on day 14 (NSD14) (Fig 6). The thickened neo-epidermis was flattened and normalised quicker in ESD14 samples compared to NSD14 tissue sections. Inflammation was decreased in different dermal niches and granulation stroma was predominantly fibroid in appearance in ESD14 tissue sections compared to a myxoid stromal appearance observed in normal healing tissue sections on day 14 (NSD14).

#### Angiogenesis markers were up-regulated following electrical stimulation

**Fig 7:**
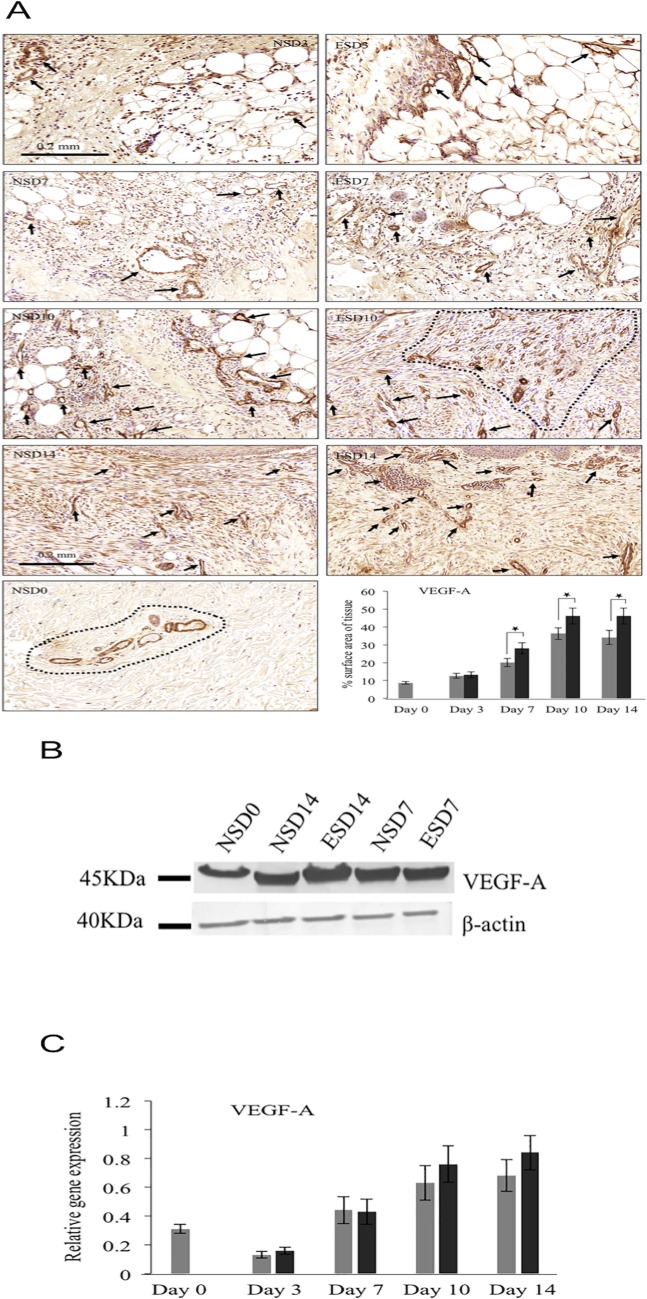
VEGF-A protein and gene analysis showed up-regulation in ES treated samples. A. Immunohistochemical analysis of VEGF-A. VEGF-A was significantly up-regulated on days 7, 10 and 14 of ES treated tissues compared to normal healing tissues of respective days. Number of vessels were also increased on day 14 ES treated tissues compared to normal healing tissues. Arrows/area inside the dotted lines indicate VEGF^+^ cells/area. B. Western blotting of VEGF-A. Protein analysis by Western blotting showed increase on day 14 ES treated tissues compared to control. C. Gene analysis of VEGF-A on different healing days showed up-regulation in ES treated tissues, compared to control. Grey bars—Normal cutaneous healing process. Black bars—Electrical stimulation assisted cutaneous healing process. * shows significant difference (p<0.05).

Expression of angiogenesis markers such as Vascular endothelial growth factor-A (VEGF-A) and Placental growth factor (PLGF) were investigated to corroborate the *in vivo* findings on differential rate of blood flow in healing tissues. Immunohistochemical staining on days 7, 10 and 14 showed a significant increase (p<0.05) in VEGF-A expression (Fig 7A) in ES-treated tissue sections (39%, 27% and 35% respectively) compared to control. Furthermore, day 3 samples did not show any significant difference in VEGF-A expression. Qualitative analysis by WB (Fig 7B) showed that there was a marginal increase in VEGF-A expression in day 14 healing samples post-ES, compared to control. Gene analysis by qRT-PCR (Fig 7C) indicated non-significant increase in ES-treated samples in all healing days.

**Fig 8:**
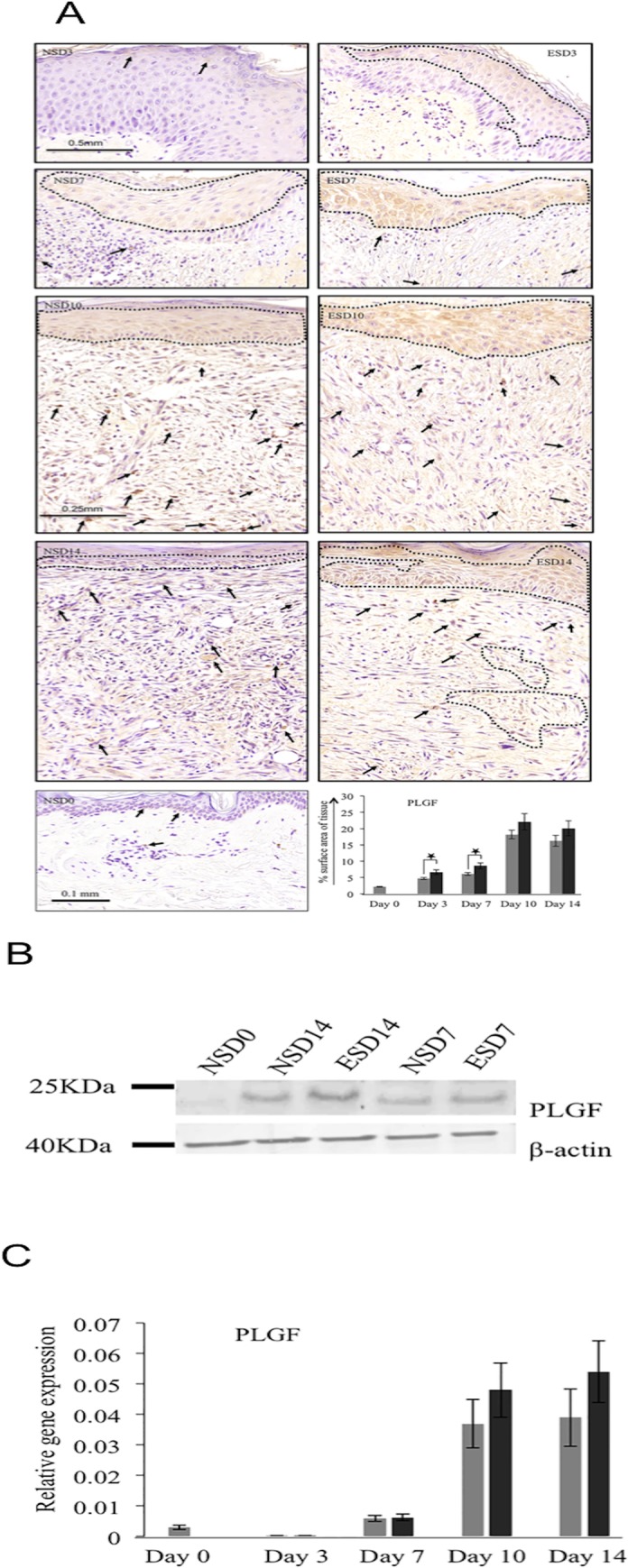
PLGF protein and gene analysis showed up-regulation in ES treated samples. A. Immunohistochemical analysis of PLGF. PLGF was significantly up-regulated on days 3 and 7 of ES treated tissues compared to control of respective days. Even though ES treated tissues showed increase of 21% and 23% on days 10 and 14 respectively when compared to control, the results were not statistically significant (p>0.05). The arrows indicate PLGF^+^ cells. B. Western blotting of PLGF. Protein analysis by Western blotting showed significant increase of PLGF on days 7 and 14 in ES treated tissues compared to normal healing tissues. C. Gene analysis of VEGF-A on different healing days showed up-regulation in ES treated tissues on days 10 and 14, compared to control. Grey bars—Normal cutaneous healing process. Black bars—Electrical stimulation assisted cutaneous healing process. * shows significant difference (p<0.05).

Further upon IHC analysis, expression of PLGF (Fig 8A) was significantly increased (p<0.05) in ES-treated tissues on days 3 and 7 (40% in both days) compared to control. Additionally, qualitative WB analysis for PLGF showed an increase on days 7 and 14 (Fig 8B) in ES-treated tissues compared to control. In the initial days of wound healing (days 0–7), PLGF was mainly expressed by the migrating keratinocytes. However, gene analysis (Fig 8C) indicated non-significant increase in ES-treated samples on days 10 and 14, compared to control samples.

## Discussion

This multiple sequential biopsy study has demonstrated significant beneficial effects of ES in enhancing acute cutaneous wound healing. We have shown that wound volume was significantly reduced following ES on day 7. Additionally, volume, surface area and diameter of the wounds were significantly reduced post-ES on days 10 and 14. Furthermore, we noted a statistically significant increase in blood flow on days 10 and 14 post-ES. This particular finding was further verified with a simultaneous up regulation of angiogenic biomarkers in wound biopsies. The EF present at the wound edges was increased following ES in the initial days of healing albeit decreased in the later stages, compared to the control. To our knowledge, this detailed study, is unique in investigating the role of ES in enhancing cutaneous wound healing in human skin. Multiple sequential time points were correlated with a number of objective parameters including histological markers to demonstrate the role of an exogenous asymmetric biphasic current in positively impacting the rate of progression of cutaneous wound healing.

The benefits of this study were the relatively larger sample size of 40 compared to our previously published study of twenty subjects [18], the inclusion of sequential temporal biopsies, randomisation to account for handedness and the utilisation of multiple non-invasive measuring devices to monitor the progression of wound healing. In the previous study, we did not utilise a device to measure the size of the wounds, therefore in the current study the use of a 3D camera proved invaluable as it enabled quantitative measurements of wound volume, surface area and diameter before and after application of ES. Additionally, for the first time, we used Dermacorder to measure the EF at the wound edges. The homogeneity of the cohorts was a strength of the study as all subjects were of a similar Fitzpatrick skin type, all were of same ethnic origin (Caucasian), all were right handed and all were of a similar age group (under 30 years of age). The participant arms were randomised to account for handedness as their dominant hand may have affected the healing of the wound, as this arm would be most active arm and in constant use. Additionally, the Fitzpatrick score gave a range of similar skin types for the participants in relation to having a homogenous cohort of subjects for melanin level measurement pre and post ES treatment. Furthermore, a single researcher conducted the treatments and data collections; therefore, inter-observer biases and errors were reduced. Evidence of wound crusting did not cause a problem when measuring the biopsy wounds. By day 7, the majority of crusting was not present with contracting 5mm biopsy wounds, therefore there was either none or minimal crusting present.

We demonstrated that the wound dimensions reduced following ES treatment compared to the controls, which was verified objectively and quantitatively by 3D imaging that calculated the surface area, volume, diameter and depth of the wound sites. This technique has also been used in other studies [28–31]. Contraction of the wound begins soon after wounding and peaks at 2 weeks [32]. The degree of wound contraction varies with the depth of the wound. For full-thickness wounds as in our study, contraction is an important part of healing and accounts for up to a 40% decrease in the size of the wound [32]. In this study, the greatest reductions following ES were for wound volume on days 7, 10 and 14 and this reduced by 38.9%, 37.9% and 44.1% respectively.

We used the Dermacorder to measure the EF at the wound edges and demonstrated that the EF was increased following the application of ES. We noted that the potential difference between the wound and the healthy skin was increased on the initial days of healing post-ES treatment. EF = Potential difference / Distance. On day 3, there was a significant reduction in the diameter of the wounds post-ES, while the EF was increased. This shows that the potential difference was increased post-ES treatment. However, on days 10 and 14 the combined effect of both reduction in wound diameter and increase in potential difference may have resulted in a greater EF post-ES treatment. It is thought that current of injury decreases with subsequent time points in wound healing [33]. On day 60, EF was reduced following ES, while the wound diameter remained similar compared to the normal healing process. This demonstrates that there is a greater reduction in potential difference following ES application in the later stages of wound healing, which indicates accelerated repair. This also indicates that a non-leaky epidermal barrier had been established faster post-ES treatment.

Nuccitelli et al [26] developed this new approach, which does not require any electrode contact at the wound site therefore making it possible to quantitate the EF near skin wounds [25,26]. The endogenous EF generated near skin wounds is of interest because keratinocyte migration and wound healing are strongly influenced by EF [26]. A previous study on the subject of galvanotaxis involving human keratinocytes indicated that the mean translocation velocity of cells were proportional to the EF [34]. Additionally, during the maturation and remodeling phase of wound healing, the epidermis re-established its structure and resistance [26]. Thus, the wound current and EF would approach to normal or control levels. The epidermal lateral EF decreases gradually with stratification in re-epithelialisation [26]. This correlates with the results observed here.

In this study, we also showed that there may be a direct effect of ES current through the skin on blood flow. We demonstrated that blood flow was significantly increased on days 10 and 14 following the application of ES. Our results are further corroborated by the findings from our previous study [18], where we showed that blood flow was significantly increased at day 14 (p = 0.027). These results were further supported by the previous gene and protein studies [19], which showed that ES significantly up-regulated angiogenesis and down-regulated inflammation on day 14. However, to validate the results in the current study, we further investigated VEGF-A and PLGF mitogens. VEGF-A is involved in the normal physiological development of blood vessels [35] and its biological effects on maturing endothelium usually takes place over the course of minutes to days during angiogenesis in wound healing. This phenomenon includes VEGF receptor phosphorylation, existing vascular dilation and permeability, and activation of endothelial cell precursors [36–39]. PLGF is also a member of the VEGF family [40] and has been shown to have strong angiogenic properties in mice [41]. However, it has been shown to be up-regulated in the active angiogenic phase of wound healing in both migrating keratinocytes and endothelial cells of blood vessels within the human wound bed [42].

The increasing vascularisation over time, associated with multiple signs of active neo-angiogenesis in the dermal microvasculature indicated that the quiescent endothelium was responsive to ES, which activated potent angiogenic mitogens [43,44]. However, the developing vascular phenotype of human dermis following ES and controls, had no distinct features, other than an increase in capillary/vessel number when observed in ES treated tissues. This striking angiogenic phenotype induced by up-regulated VEGF-A and PLGF raises the possibility that these cytokines could be further modulated exogenously when ES is given to enhance neo-angiogenesis for therapeutic purposes.

We noted an association between blood flow and wound diameter, volume and surface area post-ES on days 10 and 14, compared to the control. Blood flow was increased whilst wound dimensions decreased following treatment with ES. It has been shown in a previous study using transcutaneous electrical nerve stimulation in patients with peripheral arterial disease that capillary density was increased following treatment at 3 and 6 weeks [45]. Additionally, an increase in transcutaneous oxygen measurements were reported at the same time points. Therefore, it may be possible that ES could enhance the formation of capillaries. Follow-up time points on days 60 and 90 were used in both the current study (Cohort 1) and the previous trial [18]. The results from both studies were compared, looking at the data produced from the common measurement device, FLPI, measuring blood flow. There were some similarities between the results from both studies. However, there was a reduction in blood flow by day 90 that was shown in both studies.

The ES device used forms part of a biofeedback link with the individual’s normal physiological repair. This modality follows the theory that the normal electrical potential of skin forms a global electrical network, which reflects the underlying neurological activity through changes in skin impedance. The treatment was well tolerated by all participants and was relatively easy to use in clinical practice.

Limitations of this study include, the total number of time points employed for evaluation and performing sequential biopsies. In addition, the potential use of additional tools and objective devices for monitoring the process of repair non-invasively may add further detail and value to this study. Future studies are required to note the long-term effect of delivering ES to acute wounds beyond the day 14 time point and whether there is a need for additional treatments to be given beyond the time points discussed in this study.

## Conclusions

This study indicates that ES accelerates acute cutaneous wound healing evidenced by a reduction in wound volume, diameter and surface area and an increase in blood flow. There is clear evidence from invasive and non-invasive modalities that treatment with ES resulted in increased angiogenesis. This study further substantiates the role of ES in enhancing cutaneous wound repair evidenced by quantifiable objective measures and histological analysis observed in multiple time points. Furthermore, this treatment may have potential application for treatment of delayed and chronic wounds.

## Supporting Information

S1 Table Inclusion and exclusion criteria. Table displaying the inclusion and exclusion criteria for assessing participant suitability to be enrolled onto this study.
(DOCX)

S2 Table Demographic data. Table outlining the demographic data for the two cohorts combined.
(DOCX)

S3 Table Details of primers used for the study. (DOCX)

S4 Table List of antibodies used for immunohistochemistry analysis. Primary antibodies, secondary antibodies, concentration of antibodies, incubation parameters and detection methods used for immunohistochemistry analysis.
(DOCX)

S5 Table List of antibodies used for Western blotting. Primary antibodies, secondary antibodies, concentration of antibodies, incubation parameters and detection methods used for Western blotting.
(DOCX)

S6 Table Wound surface area data. Table displaying the data for wound surface areas for both cohorts 1 and 2: Surface Area (mm^2^) Median (Range) for Biopsy Arms and Differences in Biopsy Arms. Wound surface area was significantly reduced on days 10, 14, 30, 60 and 90.
(DOCX)

S7 Table Wound volume data. Table displaying the data for wound volume for both cohorts 1 and 2: Volume (mm^3^) Median (Range) for Biopsy Arms and Differences in Biopsy Arms. Wound volume was statistically significantly reduced following degenerate wave electrical stimulation on days 7, 10 and 14.
(DOCX)

S8 Table Wound diameter data. Table displaying the data for wound diameter for both cohorts 1 and 2: Diameter (mm) Median (Range) for Biopsy Arms and Differences in Biopsy Arms. Wound diameter was statistically significantly reduced following degenerate wave electrical stimulation on days 10, 14 and 90.
(DOCX)

S9 Table Wound depth data. Table displaying the data for wound depth for both cohorts 1 and 2: Average Depth (mm) Median (Range) for Biopsy Arms and Differences in Biopsy Arms.
(DOCX)

S10 Table Wound blood flow data. Table displaying the data for wound blood flow for both cohorts 1 and 2: Full-field laser perfusion imaging (FLPI) Median (Range) of Changes and Differences of Changes in Biopsy Arms. Blood flow was statistically significantly increased on days 10 and 14 following degenerative wave electrical stimulation.
(DOCX)
