# Supplementary material for: Angiogenesis Is Induced and Wound Size Is Reduced by Electrical Stimulation in an Acute Wound Healing Model in Human Skin
Source: PLoS One. 2015 Apr 30;10(4):e0124502. doi: 10.1371/journal.pone.0124502 (PMC4415761; doi:10.1371/journal.pone.0124502)
Supplement: S1 Table — Table displaying the inclusion and exclusion criteria for assessing participant suitability to be enrolled onto this study. (DOCX) [file pone.0124502.s001.docx]

| Inclusion Criteria  **S1 Table** | Exclusion Criteria |
| --- | --- |
| Male or Female | Subjects who do not give consent or withdraw consent to take part in the study |
| Aged 16 or over | Aged less than 16 years |
| Able to understand study requirements and attend all follow-up visits | Subjects with an active skin disorder considered to adversely affect the healing of the acute wound by the investigator |
| Able to provide written consent if competent | Subjects who have a history or evidence of keloid scarring (self reported or determined by physical examination) |
| Weight between 40-150kg with a body mass index of 20-45kg/m^2^. | Subjects who are pregnant or are planning to conceive in the next 3 months |
|  | Subjects with any likely wound healing impairment due to a clinically significant medical condition such as renal, hepatic, haematological, neurological or immune disease |
|  | Malignancy – diagnosed or treated within the last 5 years |
|  | Immunosuppressive, radiation or chemotherapy within the last 3 months |
|  | Current anticoagulant therapy (e.g warfarin), systemic steroids, hormone replacement therapy or any investigational drugs taken in the previous month |
|  | Subjects with evidence of illicit drug abuse |
|  | Subjects who are known to have hepatitis B or C infection including hepatitis B surface antigen, hepatitis B core antibodies or hepatitis C antibodies |
|  | Subjects who are HIV positive |
|  | Any subject who in the opinion of the investigator is unable to fully understand the requirements of the trial, consent or who is unable to return for follow-up appointments |
|  | Any subject who becomes systemically unwell during the research process due to external study causes |
|  | Subjects involved in other studies in the past 2 months prior to day 0 |
|  | Subjects fitted with a pacemaker device |
|  |  |
