# Supplementary material for: Angiogenesis Is Induced and Wound Size Is Reduced by Electrical Stimulation in an Acute Wound Healing Model in Human Skin
Source: PLoS One. 2015 Apr 30;10(4):e0124502. doi: 10.1371/journal.pone.0124502 (PMC4415761; doi:10.1371/journal.pone.0124502)
Supplement: S2 Table — Table outlining the demographic data for the two cohorts combined. (DOCX) [file pone.0124502.s002.docx]

**S2 Table**

| COHORT 1 - Demographics |  | Frequency | Percentage (%) |
| --- | --- | --- | --- |
| Number of participants | Total | 20 | 100 |
| Gender | Male  Female | 6  14 | 30  70 |
| Age | ≤18  19-24  25-30 | 0  15  5 | 0  75  25 |
| Ethnicity | Caucasian | 20 | 100 |
| Fitzpatrick Skin Score | I  II  III  IV  V  VI | 1  9  9  1  0  0 | 5  45  45  5  0  0 |
| Handedness | Left  Right | 0  20 | 0  100 |

| COHORT 2-Demographics |  | Frequency | Percentage (%) |
| --- | --- | --- | --- |
| Number of participants | Total | 20 | 100 |
| Gender | Male  Female | 5  15 | 25  75 |
| Age | ≤18  19-24  25-30 | 1  7  12 | 5  35  60 |
| Ethnicity | Caucasian | 20 | 100 |
| Fitzpatrick Skin Score | I  II  III  IV  V  VI | 1  8  10  1  0  0 | 5  40  50  5  0  0 |
| Handedness | Left  Right | 0  20 | 0  100 |
