# Supplementary material for: Angiogenesis Is Induced and Wound Size Is Reduced by Electrical Stimulation in an Acute Wound Healing Model in Human Skin
Source: PLoS One. 2015 Apr 30;10(4):e0124502. doi: 10.1371/journal.pone.0124502 (PMC4415761; doi:10.1371/journal.pone.0124502)
Supplement: S3 Table — (DOCX) [file pone.0124502.s003.docx]

| **Gene** | **Primers (bp)**  **FP: Forward primer**  **RP: Reverse primer** | **Accession number** | **Amplicon product size (bp)** |
| --- | --- | --- | --- |
| PLGF | FP: ggctgttcccttgcttcc (18)  RP: cagacaaggcccactgct (18) | cat. no. 04686900001 | 78 |
| RPL32 | FP: gaagttcctggtccacaacg (20)  RP: gagcgatctcggcacagta (19) | NM_000994.3 | 77 |
| VEGF-A | FP: tgcccgctgctgtctaat (18)  RP: tctccgctctgagcaagg (18) | cat. no. 04684974001 | 70 |

**S3 Table**
