# Supplementary material for: Angiogenesis Is Induced and Wound Size Is Reduced by Electrical Stimulation in an Acute Wound Healing Model in Human Skin
Source: PLoS One. 2015 Apr 30;10(4):e0124502. doi: 10.1371/journal.pone.0124502 (PMC4415761; doi:10.1371/journal.pone.0124502)
Supplement: S4 Table — Primary antibodies, secondary antibodies, concentration of antibodies, incubation parameters and detection methods used for immunohistochemistry analysis. (DOCX) [file pone.0124502.s004.docx]

**S4 Table**

| **Primary antibody name, product code and company** | **Primary antibody raised species, isotype and concentration** | **Primary antibody incubation details** | **Secondary antibody, company,**  **concentration, incubation details** | **Detection method** |
| --- | --- | --- | --- | --- |
| VEGF-A,  Ab183100, Abcam, Cambridge, UK | Rabbit (polyclonal),  IgG, 1:75 dilution | 1 hour, room temperature | Universal antibody by Novolink ^TM^  Leica Biosystems Newcastle ltd, Newcastle Upon Tyne, UK  cat. RE7150-K  (1h room temp) | Peroxidase |
| PLGF,  Ab9542, Abcam, UK | Rabbit (polyclonal),  IgG, 1:250 dilution | 1 hour, room temperature | Universal antibody by Novolink ^TM^  (1h room temp) | Peroxidase |
