# Supplementary material for: Angiogenesis Is Induced and Wound Size Is Reduced by Electrical Stimulation in an Acute Wound Healing Model in Human Skin
Source: PLoS One. 2015 Apr 30;10(4):e0124502. doi: 10.1371/journal.pone.0124502 (PMC4415761; doi:10.1371/journal.pone.0124502)
Supplement: S6 Table — Table displaying the data for wound surface areas for both cohorts 1 and 2: Surface Area (mm2) Median (Range) for Biopsy Arms and Differences in Biopsy Arms. Wound surface area was significantly reduced on days 10, 14, 30, 60 and 90. (DOCX) [file pone.0124502.s006.docx]

**S6 Table**

| Surface Area (mm^2^) Median (Range) for Biopsy Arms and Differences in Biopsy Arms | | | | | |  |
| --- | --- | --- | --- | --- | --- | --- |
| Wound Day | N | Control Arm | Post-DW Arm | Difference of  post-DW vs. Control | p-value |  |
| 0 | 20 | - | - | - |  |  |
| 3 | 20 | 15.12 (6.71, 21.16) | 14.03 (4.09, 20.43) | -0.90 (-6.26, 5.12) | 0.117 |  |
| 7  10  14 | 20  20  20 | 11.34 (7.33, 18.94)  9.21 (3.52, 15.62)  7.70 (3.63, 12.82) | 10.96 (5.74, 17.33)  7.22 (2.72, 11.95)  4.49 (2.52, 9.82) | -0.92 (-6.07, 3.47)  -1.80 (-7.02, 0.84)  -2.39 (-7.33,-0.42) | 0.232  **0.001**  **<0.001** |  |
| 30 | 20 | 15.31 (8.09, 22.26) | 12.79 (4.36, 21.65) | -2.35 (-8.25, 2.63) | **0.004** |  |
| 60 | 19 | 14.29 (7.39, 22.01) | 9.85 (3.29, 16.99) | -3.44 (-6.90, 3.04) | **0.001** |  |
| 90 | 19 | 12.34 (6.39, 20.23) | 8.64 (2.85, 15.31) | -2.54 (-6.72, 2.45) | **0.003** |  |
| Difference: Measurements Post-DW – Measurements Control | | | | | |  |
| p-values from unadjusted paired Wilcoxon signed ranks tests, 1% significance level | | | | | | |
|  | | | | | | |
