# Supplementary material for: Angiogenesis Is Induced and Wound Size Is Reduced by Electrical Stimulation in an Acute Wound Healing Model in Human Skin
Source: PLoS One. 2015 Apr 30;10(4):e0124502. doi: 10.1371/journal.pone.0124502 (PMC4415761; doi:10.1371/journal.pone.0124502)
Supplement: S7 Table — Table displaying the data for wound volume for both cohorts 1 and 2: Volume (mm3) Median (Range) for Biopsy Arms and Differences in Biopsy Arms. Wound volume was statistically significantly reduced following degenerate wave electrical stimulation on days 7, 10 and 14. (DOCX) [file pone.0124502.s007.docx]

**S7 Table**

| Volume (mm^3^) Median (Range) for Biopsy Arms and Differences in Biopsy Arms | | | | | | |
| --- | --- | --- | --- | --- | --- | --- |
| Wound Day | N | Control Arm | Post-DW Arm | Difference of  Post-DW vs. Control | p-value |  |
| 0 | 20 | - | - | - |  |  |
| 3 | 20 | 2.29 (0.98, 4.47) | 1.55 (0.16, 5.02) | -0.38 (-2.57, 2.91) | 0.093 |  |
| 7  10  14 | 20  20  20 | 1.98 (0.81, 3.83)  2.48 (1.10,5.30)  2.04 (0.08, 4.25) | 1.21 (0.32, 4.16)  1.54 (0.64, 4.37)  1.14 (0.06, 4.86) | -0.74 (-3.26, 2.27)  -0.60 (-3.94, 1.11)  -0.93 (-2.89, 0.61) | **0.003**  **0.002**  **<0.001** |  |
| 30 | 20 | 3.06 (1.35, 8.96) | 2.50 (0.28, 8.34) | -0.77 (-3.90, 1.85) | 0.030 |  |
| 60 | 19 | 2.84 (1.08, 5.92) | 1.87 (0.25, 5.48) | -0.60 (-2.61, 1.84) | 0.030 |  |
| 90 | 19 | 2.13 (1.05, 4.07) | 1.38 (0.07, 4.87) | -0.47 (-2.24, 1.31) | 0.133 |  |
| Difference: Measurements Post-DW– Measurements Control | | | | | | |
| p-values from unadjusted paired Wilcoxon signed ranks tests, 1% significance level | | | | | |  |
|  | | | | | |  |
