# Supplementary material for: Angiogenesis Is Induced and Wound Size Is Reduced by Electrical Stimulation in an Acute Wound Healing Model in Human Skin
Source: PLoS One. 2015 Apr 30;10(4):e0124502. doi: 10.1371/journal.pone.0124502 (PMC4415761; doi:10.1371/journal.pone.0124502)
Supplement: S8 Table — Table displaying the data for wound diameter for both cohorts 1 and 2: Diameter (mm) Median (Range) for Biopsy Arms and Differences in Biopsy Arms. Wound diameter was statistically significantly reduced following degenerate wave electrical stimulation on days 10, 14 and 90. (DOCX) [file pone.0124502.s008.docx]

**S8 Table**

| Diameter (mm) Median (Range) for Biopsy Arms and Differences in Biopsy Arms | | | | | | |
| --- | --- | --- | --- | --- | --- | --- |
| Wound Day | N | Control Arm | Post-DW Arm | Difference of  Post-DW vs. Control | p-value | |
| 0 | 20 | - | - | - |  | |
| 3 | 20 | 5.14 (3.95, 6.19) | 5.05 (3.65, 5.81) | -0.16 (-0.96, 0.69) | 0.083 | |
| 7  10  14 | 20  20  20 | 4.72 (3.42, 5.64)  4.32 (2.56, 5.43)  3.75 (2.68, 5.26) | 4.58 (3.23, 5.54)  3.84 (2.57, 5.11)  3.15 (2.42, 5.56) | -0.25 (-1.75, 1.05)  -0.35 (-1.90, 0.64)  -0.56 (-1.62, 1.16) | 0.225  **0.009**  **0.002** | |
| 30 | 20 | 5.86 (3.27, 7.25) | 5.71 (3.89, 6.61) | -0.13 (-2.02, 1.87) | 0.550 | |
| 60 | 19 | 4.99 (3.13, 6.94) | 4.76 (2.13, 6.71) | -0.23 (-1.94, 1.01) | 0.103 | |
| 90 | 19 | 4.39 (2.75, 5.88) | 3.68 (2.01, 5.40) | -0.71 (-1.76, 0.70) | **0.007** | |
| Difference: Measurements Post-DW – Measurements Control | | | | | | |
| p-values from unadjusted paired Wilcoxon signed ranks tests, 1% significance level | | | | | |  |
|  | | | | | |  |
