# Supplementary material for: Angiogenesis Is Induced and Wound Size Is Reduced by Electrical Stimulation in an Acute Wound Healing Model in Human Skin
Source: PLoS One. 2015 Apr 30;10(4):e0124502. doi: 10.1371/journal.pone.0124502 (PMC4415761; doi:10.1371/journal.pone.0124502)
Supplement: S9 Table — Table displaying the data for wound depth for both cohorts 1 and 2: Average Depth (mm) Median (Range) for Biopsy Arms and Differences in Biopsy Arms. (DOCX) [file pone.0124502.s009.docx]

**S9 Table**

| Average Depth (mm) Median (Range) for Biopsy Arms and Differences in Biopsy Arms | | | | | | |
| --- | --- | --- | --- | --- | --- | --- |
| Wound Day | N | Control Arm | Post-DW Arm | Difference of  Post-DW vs. Control | p-value | |
| 0 | 20 | - | - | - |  | |
| 3 | 20 | 0.27 (0.01, 1.37) | 0.25 (0.04, 1.30) | -0.07 (-0.55, 0.32) | 0.260 | |
| 7  10  14 | 20  20  20 | 0.27 (0.02, 1.60)  0.50 (0.10, 1.20)  0.36 (0.11, 1.12) | 0.22 (0.02, 1.20)  0.39 (0.15, 1.10)  0.31 (0.03, 0.75) | -0.06 (-0.73, 0.24)  -0.01 (-0.85, 0.83)  -0.05 (-0.81, 0.63) | 0.048  0.390  0.341 | |
| 30 | 20 | 0.34 (0.10, 1.20) | 0.34 (0.08 ,1.60) | 0.05 (-1.12, 0.63) | 0.732 | |
| 60 | 19 | 0.26 (0.10, 1.40) | 0.20 (0.06, 0.64) | -0.12 (-1.30, 0.22) | 0.040 | |
| 90 | 19 | 0.24 (0.11, 1.10) | 0.19 (0.02, 0.52) | -0.11 (-0.93, 0.16) | 0.014 | |
| Difference: Measurements Post-DW – Measurements Control | | | | | | |
| p-values from unadjusted paired Wilcoxon signed ranks tests, 1% significance level | | | | | |  |
|  | | | | | |  |
