# Supplementary figures and images for: Angiogenesis Is Induced and Wound Size Is Reduced by Electrical Stimulation in an Acute Wound Healing Model in Human Skin
Source: PLoS One. 2015 Apr 30;10(4):e0124502. doi: 10.1371/journal.pone.0124502 (PMC4415761; doi:10.1371/journal.pone.0124502)

**S10 Table**

**
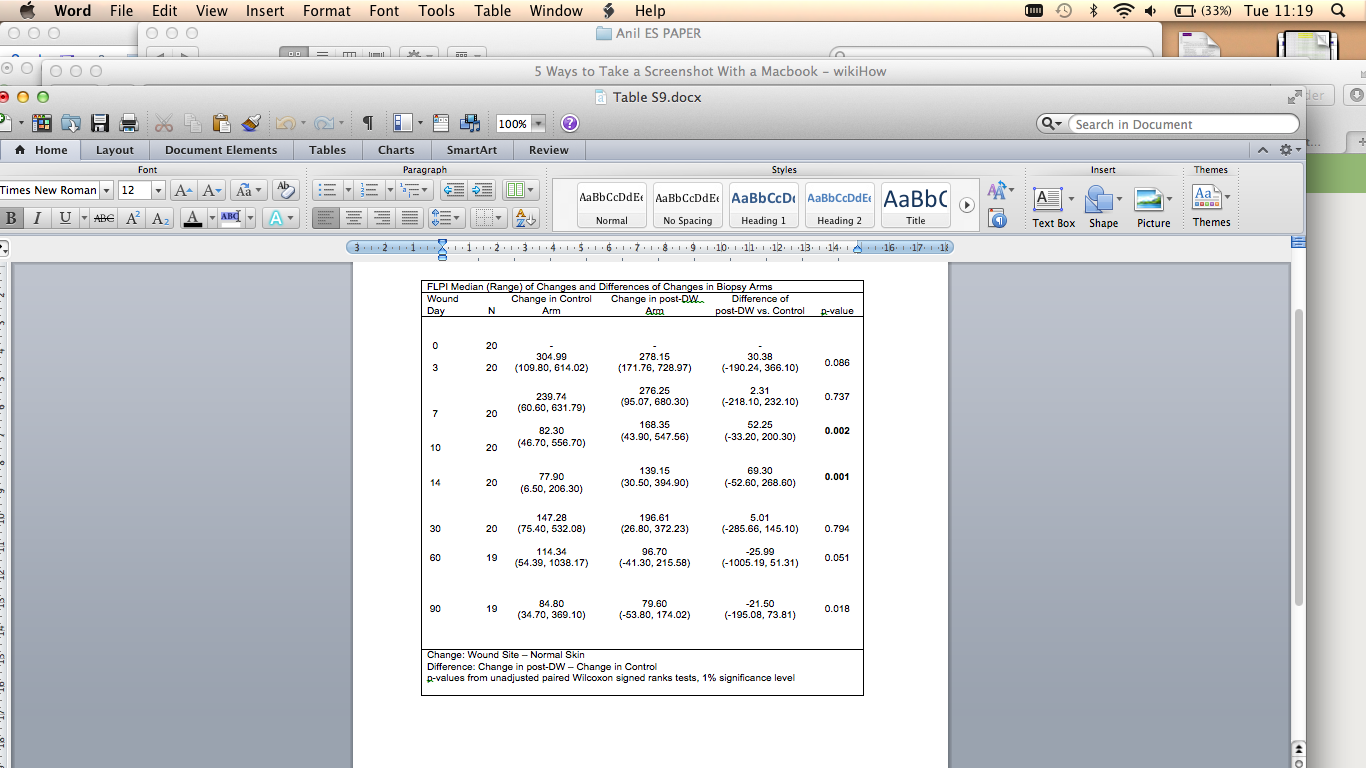
**

Supplement: S10 Table — Table displaying the data for wound blood flow for both cohorts 1 and 2: Full-field laser perfusion imaging (FLPI) Median (Range) of Changes and Differences of Changes in Biopsy Arms. Blood flow was statistically significantly increased on days 10 and 14 following degenerative wave electrical stimulation. (DOCX) [file pone.0124502.s010.docx]
